# Persistent misconceptions about HIV transmission among males and females in Malawi

**DOI:** 10.1186/s12914-016-0089-8

**Published:** 2016-06-07

**Authors:** Yujiro Sano, Roger Antabe, Kilian Nasung Atuoye, Lucia Kafui Hussey, Jason Bayne, Sylvester Zackaria Galaa, Paul Mkandawire, Isaac Luginaah

**Affiliations:** Department of Sociology, University of Western Ontario, 1151 Richmond Street, London, ON Canada; Environmental Health and Hazards Lab, Department of Geography, University of Western Ontario, 1151 Richmond Street, London, ON Canada; Faculty of Integrated Development Studies, University for Development Studies, Wa Campus, Wa, Ghana; Institute of Interdisciplinary Studies, Human Rights Program, Carleton University, Ottawa, Canada; Department of Geography, University of Western Ontario, 1151 Richmond Street, London, ON Canada

**Keywords:** HIV/AIDS Misconceptions, Gender, Malawi, Sub-Saharan Africa

## Abstract

**Background:**

The prevalence of HIV in Malawi is one of the highest in sub-Saharan Africa, and misconceptions about its mode of transmission are considered a major contributor to the continued spread of the virus.

**Methods:**

Using the 2010 Malawi Demographic and Health Survey, the current study explored factors associated with misconceptions about HIV transmission among males and females.

**Results:**

We found that higher levels of ABC prevention knowledge were associated with lower likelihood of endorsing misconceptions among females and males (OR = 0.85, *p* < 0.001; OR = 0.85, *p* < 0.001, respectively). Compared to those in the Northern region, both females and males in the Central (OR = 0.54, *p* < 0.001; OR = 0.53, *p* < 0.001, respectively) and Southern regions (OR = 0.49, *p* < 0.001; OR = 0.43, *p* < 0.001, respectively) were less likely to endorse misconceptions about HIV transmission. Moreover, marital status and ethnicity were significant predictors of HIV transmission misconceptions among females but not among males. Also, household wealth quintiles, education, religion, and urban–rural residence were significantly associated with endorsing misconceptions about HIV transmission.

**Conclusion:**

Based on our findings, we recommend that education on HIV transmission in Malawi should integrate cultural and ethnic considerations of HIV/AIDS.

## Background

Malawi is the world’s ninth worst HIV-affected country, with an adult prevalence of 10.6 %, nearly twice higher than the sub-Saharan African (SSA) regional average of 5.7 % [1]. To address the high HIV prevalence, Malawi adopted the National HIV Prevention Strategy, with its aim to reduce new HIV infections and mitigate the burden and impact of HIV [2]. One major thrust of this strategic plan is behavioural change, which assumes that individuals are likely to reduce risky behaviours once they understand the modes of HIV transmission [2]. Thus, the main objective of this strategy is to increase ABC knowledge of HIV prevention (abstinence, being faithful, and condom use).

Despite major efforts at behaviour change, myths and misconceptions about HIV transmission remain an important influence on risky sexual behaviours [3–6]. Subsequently, the behavioural change model is limited in capturing the influence of myths and misconceptions to inform clinical or scientific understanding of the ways in which HIV is transmitted or prevented [7, 8]. Myths and misconceptions may refer to ideas and concepts believed or held by a group of people, which are scientifically proven to be untrue [9–11]. Given that these two concepts are mostly used interchangeably in the literature (see [11, 12]), we have also used them accordingly in this paper.

Misconceptions about HIV transmission are still prevalent in SSA, despite recent significant improvements in public awareness of the disease. For example, studies in Kenya and Ghana discovered that some respondents believe the HIV virus could be spread through mosquito bites and touching an infected person [11, 13]. In addition, the view that a healthy-looking person cannot be HIV positive or that HIV can be cured by sleeping with a virgin, eating fresh vegetables, and making ancestral sacrifices is still a notion in many parts of SSA [14, 15].

Studies also suggest that such misconceptions are a result of the interaction between individual and societal influences [16, 17]. At the individual level, lack of ABC prevention knowledge has been considered an important explanation for misconceptions about HIV [11, 18]. This is consistent with an empirical finding that people with a clear understanding of biological processes of HIV transmission are more likely to reject such misconceptions [19]. This indicates that clinical knowledge about HIV transmission is critical in eliminating HIV-related misconceptions. Moreover, other studies indicate that individuals who perceive themselves as more vulnerable to HIV/AIDS are less likely to endorse misconceptions about HIV. Thus, such individuals are likely to seek more information about the disease [20]. Also, clinical knowledge gained during counselling before testing is useful in increasing awareness about HIV transmission [21].

Misconceptions about HIV transmission are found to have cultural undertones. In Malawi, diseases such as “tsempho” and “kanyera” are believed to result from sexual promiscuity and indulging in sex-related taboos. These diseases share similar symptoms as HIV infection, such as marasmic appearance, generalized edema, weight loss, and diarrhea [22]. Thus, understanding the etiology of HIV and other sexually transmitted infections (STIs) among some cultural groups could be different from clinical and biological explanations. While misconceptions about HIV transmission may be widespread among a population, there may be ethnic variations in endorsing such misconceptions, given that some ethnic groups are more likely to preserve cultural and ethnic interpretations of the disease [23, 24].

Moreover, ethnic variations in endorsing misconceptions may be different between women and men. Although the prevalence of HIV infection among males and females in Malawi does not show any striking differences [25], the feminization of the disease in the predominantly patriarchal society may also reinforce myths and misconceptions about HIV transmission [22, 26]. This is evident in the description of STIs as “woman’s disease” in the National Chichewa language [26].

In addition to ethnicity, region and religion may shape the individual understanding of HIV transmission, which may be linked to endorsing misconceptions [27]. For example, some Islamic and Christian leaders allude to HIV being a punishment from God as a way of maintaining virtue and cosmic order [28–30]. While ABC prevention knowledge is advocated by both Christianity and Islam, Muslim preachers especially emphasize strict adherence to religious moral codes, which to them are paramount in preventing HIV infection [31, 32].

Maulana et al. [33] further found that while abstinence is supported, safer sex options, such as being faithful and condom use are not openly discussed among unmarried Muslims. This may limit opportunities to learn about HIV transmission. Also, Bello et al. [34] have shown that there is an increasing trend in HIV prevalence in the Northern region of Malawi. Given that increasing HIV prevalence could be due to high degree of misconceptions about HIV transmission, regional disparities in HIV prevalence may be linked to different patterns in endorsing such misconceptions.

Socioeconomic factors also influence the endorsement of misconceptions about HIV transmission. In Ghana, Tenkorang [11] found that wealthier and more educated Ghanaians were less likely to endorse such misconceptions than their poorer and less educated counterparts. Similarly, in Botswana, higher levels of education are linked to lower likelihood of endorsing such misconceptions [35]. These results are attributed to the fact that educated and wealthy individuals have more exposure to awareness and knowledge about HIV transmission.

Earlier research indicates that endorsing misconceptions about HIV transmission is linked to risky sexual behaviours, including early sexual debut, non-use of contraceptive methods, and inability to negotiate for safer sexual intercourse [3–5]. Despite its association with risky sexual behaviours, which could lead to HIV transmission, there are only few studies that examine the prevalence and factors associated with misconceptions about HIV transmission [11, 35]. It is our aim to add to the literature by exploring what makes Malawian women and men endorse misconceptions about HIV transmission.

## Methods

### Data and analytical sample

We used data from the 2010 Malawi Demographic and Health Survey (MDHS), a nationally representative dataset collected by the National Statistical Office in partnership with the Ministry of Health Community Sciences Unit, and the fourth of its kind to be conducted in Malawi as part of the worldwide MEASURE DHS project. The MDHS provides high quality and reliable information on basic demographic indices and knowledge and behaviours related to HIV, including misconceptions about HIV transmission. The MDHS identified 23,748 women (aged 15–49 years) and 7,783 men (aged 15–54 years) from a total of 27,000 households. Out of these participants, 23,020 women and 7,175 men were successfully interviewed, yielding a response rate of 97 % and 92 % for women and men, respectively [1]. For the purpose of this study, the sample was limited to 22,489 women and 6,978 men, who answered all the questions about HIV transmission beliefs.

### Measures

The construction of our dependent variable followed other studies that defined “misconceptions about HIV transmission” as common concepts and ideas that are widely accepted in society yet are without clinical and scientific basis [9–11, 35]. There were four questions related to HIV transmission beliefs: whether respondents believe that 1) HIV can be spread through witchcraft and other supernatural means; 2) HIV can be spread through sharing food with a person with HIV; 3) HIV can be spread through mosquito bites; 4) and, a healthy-looking person cannot be infected with HIV. Previous studies [3–6] have concluded that endorsing any form and/or number of misconception about HIV transmission is associated with risky sexual behaviours. Also, governments and NGOs in Malawi have put much effort into eliminating misconceptions about HIV transmission [2]. To reflect these positions, we constructed a binary dependent variable. Specifically, if respondents reported “yes” to at least any one of the four questions, they were categorized as endorsing misconceptions about HIV transmission (0 = not endorsing misconceptions; 1 = endorsing misconceptions).

Independent variables were grouped into three blocks: behavioural, demographic, and socioeconomic factors. Behavioural factors include two variables, ABC prevention knowledge and whether respondents have been tested for HIV. Using Principal Component Analysis (PCA), a summative scale called “ABC prevention knowledge” was constructed from three variables: whether respondents were knowledgeable about the following HIV prevention strategies: 1) abstinence; 2) being faithful; and, 3) condom use (0 = no; 1 = yes). All items loaded on one latent construct, with factor loadings ranging between 0.66 and 0.74. Cronbach’s alpha was estimated at 0.53 and 0.48 for women and men, respectively. Positive values indicate higher levels of ABC prevention knowledge while negative values mean lower levels of knowledge. For demographic variables, we included ethnicity (0 = Tumbuka; 1 = Chewa; 2 = Lomwe; 3 = Tonga; 4 = Yao; 5 = Sena; 6 = Ngori; 7 = other), religion (0 = Christian; 1 = Muslim; 2 = no religion), region of residence (0 = Northern; 1 = Central; 2 = Southern), urban–rural residence (0 = urban; 1 = rural), marital status (0 = never married; 1 = currently married; 2 = formerly married), and age of respondents (measured in completed years). Finally, there were three variables linked to socioeconomic status, such as educational background (0 = no formal education; 1 = primary education; 2 = secondary/higher education), household wealth quintiles (0 = poorest; 1 = poorer; 2 = middle; 3 = richer; 4 = richest), and whether respondents were working (0 = no; 1 = yes).

### Analytic technique

Due to the dichotomous nature of the dependent variable (misconceptions about HIV transmission), we employed logistic regression analysis. Also, we ran six multivariate models, three models each for men and women. The results were reported in the form of odds ratios. Whereas odds ratios greater than one indicate higher likelihood of holding misconceptions about HIV transmission, odds ratios of less than one indicate lower likelihood of holding such misconceptions. Also, due to the multistage sample design and complex population-based surveys such as the MDHS, there is usually some degree of dependence among the observations. Given that the assumption of independence is required for estimating accurate standard errors, we addressed this potential bias by accounting for clustering, which adjusted the standard errors and produced statistically robust parameter estimates [36].

## Results

### Univariate analyses

Table 1 shows the results from univariate analyses of misconceptions about HIV transmission and selected independent variables. A larger proportion of women (44.6 %) endorsed at least one of the misconceptions about HIV transmission than their male counterparts (37.2 %). Also, 74.7 % and 54 % of women and men, respectively, reported being tested for HIV. Over 80 % of all respondents lived in the Southern region, resided in rural areas and were Christian. The largest ethnic group in the selected sample was Chewa, accounting for 29.4 % of respondents, followed by Lomwe, Ngori, and Yao ethnic groups. While household wealth quintiles were evenly spread among women and men, more men (29.6 %) than women (18.9 %) had attained secondary or higher education. Also, fewer women (56.9 %) were working compared to men (81.9 %).

**Table 1:** Univariate analysis of selected dependent and independent variables

	Women (%)	Men (%)
Misconceptions about HIV transmission		
Not endorsing any misconception	55.4	62.8
Endorsing at least one misconception	44.6	37.2
ABC prevention knowledge (median)	0.70	0.73
Have been tested for AIDS		
No	25.3	46.0
Yes	74.7	54.0
Region of residence		
Northern	18.2	18.0
Central	34.0	36.1
Southern	47.9	45.9
Ethnicity		
Tumbuka	10.9	10.1
Chewa	29.4	29.1
Lomwe	16.3	17.9
Tonga	3.26	3.50
Yao	10.5	10.6
Sena	5.63	5.49
Ngori	13.6	12.8
Others	10.5	10.5
Religion		
Christian	88.4	87.1
Muslim	11.0	10.3
No religion	0.59	2.68
Urban-rural residence		
Urban	13.5	14.2
Rural	86.5	85.8
Marital status		
Never married	19.6	37.7
Currently married	67.1	58.8
Formerly married	13.3	3.55
Age of respondents (mean)	28.1	29.1
Educational background		
No education	14.6	6.02
Primary education	66.6	64.4
Secondary/higher education	18.9	29.6
Wealth status		
Poorest	19.5	15.8
Poorer	19.5	20.2
Middle	20.5	20.5
Richer	20.5	21.5
Richest	20.0	22.0
Currently working		
No	43.1	18.1
Yes	56.9	81.9
Total	22,489	6,978

### Bivariate analyses

Results from bivariate analyses between misconceptions about HIV transmission and selected independent variables are shown in Table 2. Higher levels of ABC prevention knowledge were associated with lower likelihood of endorsing misconceptions about HIV transmission for women and men (OR = 0.83, *p* < 0.001; OR = 0.80, *p* < 0.001, respectively). Similarly, compared to those who had not been tested for HIV, both women (OR = 0.85, *p* < 0.001) and men (OR = 0.77, *p* < 0.001) who had been tested were less likely to endorse such misconceptions. Demographic factors were also significantly associated with endorsing misconceptions. Compared to respondents in the Northern region, those in the Central region were less likely to have misconceptions about transmission of the virus (OR = 0.67, *p* < 0.001; OR = 0.51, *p* < 0.001 for women and men, respectively). In addition, compared to respondents in the Northern region, residents in the Southern region were less likely to endorse HIV misconception (OR = 0.69, *p* < 0.001; OR = 0.57, *p* < 0.001 for women and men, respectively). We also found that both Tumbuka women and men were more likely to endorse misconceptions about HIV transmission than those in other ethnic groups.

**Table 2:** Bivariate analysis of selected dependent and independent variables

	Women		Men	
	OR	[CI 95 %]	OR	[CI 95 %]
ABC prevention knowledge	0.83***	[0.78, 0.87]	0.80***	[0.78, 0.83]
Have been tested for AIDS				
No	1.00		1.00	
Yes	0.85***	[0.80, 0.90]	0.77***	[0.70, 0.84]
Region of residence				
Northern	1.00		1.00	
Central	0.67***	[0.62, 0.72]	0.69***	[0.60, 0.79]
Southern	0.51***	[0.48, 0.54]	0.57***	[0.50, 0.65]
Ethnicity				
Tumbuka	1.00		1.00	
Chewa	1.41***	[1.27, 1.55]	1.26**	[1.07, 1.49]
Lomwe	0.69***	[0.64, 0.75]	0.83*	[0.70, 0.97]
Tonga	0.97	[0.82, 1.15]	0.99	[0.73, 1.34]
Yao	0.85**	[0.76, 0.95]	1.11	[0.96, 1.28]
Sena	0.75***	[0.65, 0.87]	0.83	[0.64, 1.08]
Ngori	0.72***	[0.66, 0.80]	0.89	[0.73, 1.08]
Others	0.99	[0.90, 1.10]	1.07	[0.88, 1.30]
Religion				
Christian	1.00		1.00	
Muslim	1.12*	[1.02, 1.22]	1.31***	[1.14, 1.50]
No religion	1.30	[0.93, 1.81]	1.28	[0.99, 1.66]
Urban–rural residence				
Urban	1.00		1.00	
Rural	2.18***	[2.02, 2.36]	2.10***	[1.77, 2.48]
Marital status				
Never married	1.00		1.00	
Currently married	1.37***	[1.27, 1.47]	1.17*	[1.04, 1.31]
Formerly married	1.25***	[1.13, 1.37]	1.24	[0.94, 1.64]
Age of respondents	1.01***	[1.01, 1.01]	1.00	[0.99, 1.01]
Educational background				
No education	1.00		1.00	
Primary education	0.72***	[0.66, 0.78]	0.49***	[0.41, 0.59]
Secondary/higher education	0.25***	[0.23, 0.28]	0.17***	[0.14, 0.21]
Wealth status				
Poorest	1.00		1.00	
Poorer	0.89**	[0.83, 0.96]	0.77***	[0.67, 0.89]
Middle	0.81***	[0.75, 0.88]	0.72***	[0.63, 0.82]
Richer	0.68***	[0.63, 0.74]	0.53***	[0.46, 0.62]
Richest	0.39***	[0.36, 0.43]	0.34***	[0.29, 0.39]
Currently working				
No	1.00		1.00	
Yes	1.05	[0.99, 1.11]	1.07	[0.99, 1.17]

* *p* < 0.05, ** *p* < 0.01, *** *p* < 0.001

Odds ratios adjusted for clustering; 95% confidence intervals in brackets

Moreover, Muslim women and men (OR = 1.12, *p* < 0.05; OR = 1.31, *p* < .001, respectively) were more likely to endorse misconceptions than their Christian counterparts. Also, those in rural areas (OR = 2.18, *p* < 0.001; OR = 2.10, *p* < 0.001 for women and men, respectively) were more likely to have misconceptions compared to those in urban areas. Compared to those who were never married, currently married and formerly married women (OR = 1.64, *p* < 0.001; OR = 1.25, *p* < 0.001, respectively) were more likely to endorse misconceptions about HIV transmission, while only currently married men (OR = 1.17, *p* < 0.05) were more likely to endorse such misconceptions. Age was positively associated with holding misconceptions about HIV transmission among women (OR = 1.01, *p* < 0.001). Moreover, socioeconomic factors were significantly associated with endorsing misconceptions about HIV transmission. Compared to those without formal education, women and men with primary education and secondary or higher education were less likely to endorse misconceptions. Furthermore, compared to poorer women and men, their wealthier counterparts are less likely to endorse misconceptions about HIV transmission.

### Multivariate analyses

Tables 3 and 4 present the net effects of independent variables on misconceptions about HIV transmission for women and men, respectively. Consistent with bivariate results, behavioural factors were significantly associated with endorsing misconceptions about HIV transmission among women and men, even after controlling for demographic and socioeconomic factors. Malawian women and men with higher levels of ABC prevention knowledge were less likely to endorse misconceptions compared to those with lower levels of such knowledge (OR = 0.85, *p* < 0.001; OR = 0.85, *p* < 0.001, respectively). Similarly, both women and men who had been tested for HIV were less likely to have misconceptions compared to those who had not (OR = 0.85, *p* < 0.001; OR = 0.88, *p* < 0.01, respectively). While most demographic variables were significant at the bivariate level, the introduction of socioeconomic variables attenuated the effect of some demographic variables. This was particularly the case with men (see Models 2 and 3 of Tables 3 and 4). For women, those in the Central and Southern regions were less likely to endorse misconceptions about HIV transmission compared to those in the Northern region (OR = 0.54, *p* < 0.001 and OR = 0.49, *p* < 0.001, respectively).

**Table 3:** Multivariate analysis of misconceptions about HIV transmission among women in Malawi

	Model 1		Model 2		Model 3	
	OR	[95 % CI]	OR	[95 % CI]	OR	[95 % CI]
ABC prevention knowledge	0.81***	[0.79, 0.83]	0.83***	[0.81, 0.86]	0.85***	[0.83, 0.87]
Have been tested for AIDS						
No	1.00		1.00		1.00	
Yes	0.88***	[0.83, 0.92]	0.76***	[0.71, 0.82]	0.85***	[0.79, 0.91]
Region of residence						
Northern			1.00		1.00	
Central			0.59***	[0.51, 0.68]	0.54***	[0.47, 0.63]
Southern			0.53***	[0.47, 0.59]	0.49***	[0.43, 0.55]
Ethnicity						
Tumbuka			1.00		1.00	
Chewa			1.01	[0.86, 1.18]	0.83*	[0.70, 0.98]
Lomwe			0.85	[0.74, 0.99]	0.75***	[0.64, 0.88]
Tonga			0.68***	[0.58, 0.80]	0.63***	[0.54, 0.74]
Yao			0.80*	[0.67, 0.96]	0.69***	[0.57, 0.83]
Sena			0.88	[0.72, 1.08]	0.67***	[0.54, 0.80]
Ngori			0.83**	[0.72, 0.96]	0.71***	[0.61, 0.83]
Others			0.86*	[0.76, 0.98]	0.73***	[0.64, 0.82]
Religion						
Christian			1.00		1.00	
Muslim			1.34***	[1.18, 1.51]	1.22***	[1.08, 1.38]
No religion			1.24	[0.90, 1.72]	1.04	[0.76, 1.44]
Urban–rural residence						
Urban			1.00		1.00	
Rural			2.03***	[1.86, 2.22]	1.29***	[1.16, 1.42]
Marital status						
Never married			1.00		1.00	
Currently married			1.40***	[1.26, 1.55]	1.12*	[1.01, 1.25]
Formerly married			1.29***	[1.13, 1.47]	1.00	[0.88, 1.13]
Age of respondents			1.01***	[1.00, 1.02]	1.00	[0.99, 1.00]
Educational background						
No education					1.00	
Primary education					0.70***	[0.65, 0.77]
Secondary/higher education					0.30***	[0.27, 0.33]
Wealth status						
Poorest					1.00	
Poorer					0.89**	[0.82, 0.96]
Middle					0.83***	[0.76, 0.91]
Richer					0.75***	[0.68, 0.82]
Richest					0.61***	[0.55, 0.67]
Currently working						
No					1.00	
Yes					1.05	[0.99, 1.12]
Total	22,489		22,489		22,489	
Log Pseudo-likelihood	−15,312		−14,903		−14,505	

* *p* < 0.05, ** *p* < 0.01, *** *p* < 0.001

Odds ratios adjusted for clustering; 95% confidence intervals in brackets

**Table 4:** Multivariate analysis of misconceptions about HIV transmission among men in Malawi

	Model 1		Model 2		Model 3	
	OR	[95 % CI]	OR	[95 % CI]	OR	[95 % CI]
ABC prevention knowledge	0.83***	[0.79, 0.88]	0.84***	[0.79, 0.89]	0.85***	[0.80, 0.90]
Have been tested for AIDS						
No	1.00		1.00		1.00	
Yes	0.78***	[0.71, 0.86]	0.74***	[0.68, 0.82]	0.88**	[0.80, 0.96]
Region of residence						
Northern			1.00		1.00	
Central			0.57***	[0.46, 0.71]	0.53***	[0.43, 0.65]
Southern			0.47***	[0.37, 0.60]	0.43***	[0.35, 0.54]
Ethnicity						
Tumbuka			1.00		1.00	
Chewa			1.14	[0.89, 1.47]	0.94	[0.73, 1.21]
Lomwe			1.18	[0.91, 1.54]	1.06	[0.81, 1.40]
Tonga			0.80	[0.61, 1.05]	0.80	[0.61, 1.05]
Yao			1.18	[0.84, 1.66]	1.10	[0.76, 1.57]
Sena			1.20	[0.82, 1.77]	1.03	[0.70, 1.52]
Ngori			1.17	[0.85, 1.62]	1.07	[0.77, 1.49]
Others			1.08	[0.87, 1.33]	0.94	[0.75, 1.17]
Religion						
Christian			1.00		1.00	
Muslim			1.49***	[1.21, 1.84]	1.22	[0.98, 1.53]
No religion			1.15	[0.88, 1.52]	0.91	[0.69, 1.21]
Urban–rural residence						
Urban			1.00		1.00	
Rural			2.02***	[1.70, 2.41]	1.22*	[1.01, 1.48]
Marital status						
Never married			1.00		1.00	
Currently married			1.28**	[1.07, 1.52]	1.14	[0.95, 1.37]
Formerly married			1.33	[0.96, 1.83]	1.06	[0.77, 1.46]
Age of respondents			1.00	[0.99, 1.00]	0.99	[0.99, 1.00]
Educational background						
No education					1.00	
Primary education					0.51***	[0.43, 0.61]
Secondary/higher education					0.21***	[0.17, 0.26]
Wealth status						
Poorest					1.00	
Poorer					0.84*	[0.74, 0.96]
Middle					0.79***	[0.69, 0.90]
Richer					0.66***	[0.56, 0.77]
Richest					0.58***	[0.51, 0.67]
Currently working						
No					1.00	
Yes					1.02	[0.91, 1.15]
Total	6,978		6,978		6,978	
Log Pseudo-likelihood	−4,563		−4,463		−4,289	

* *p* < 0.05, ** *p* < 0.01, *** *p* < 0.001

Odds ratios adjusted for clustering; 95% confidence intervals in brackets

We also observed that the impact of cultural factors, such as ethnicity and religion, were no longer significant for men, while they remained significant for women. Compared to Christian women, their Muslim counterparts were more likely to endorse misconceptions about HIV transmission (OR = 1.22, *p* < 0.001). Meanwhile, compared to Tumbuka women, women from other ethnic groups were less likely to endorse such misconceptions. It is noteworthy that after controlling for socioeconomic factors, the gaps between Tumbuka women and women from other ethnic groups became wider (see Models 2 and 3 of Table 3). Further analysis indicated that educational background suppressed the impact of ethnicity on misconceptions about HIV transmission. We also ran a cross-tabulation between ethnicity and education and found that Tumbuka women were more educated than women from other ethnic groups.

Moreover, women in rural areas and currently married women were more likely to endorse misconceptions than their urban and never married counterparts (OR = 1.29, *p* < 0.001; OR = 1.12, *p* < 0.05, respectively). For men, only region of residence and urban–rural residence were significant. While men residing in the Central and Southern regions were less likely to endorse misconceptions about HIV transmission compared to those in Northern region (OR = 0.53, *p* < 0.001 and OR = 0.43, *p* < 0.001, respectively), men in rural areas (OR = 1.22, *p* < 0.05) were more likely to endorse such misconceptions compared to those in urban areas. In terms of socioeconomic status, we found that more educated and wealthier women and men were less likely to endorse misconceptions about HIV transmission than their less educated and poorer counterparts (see Model 3 of Tables 2 and 3).

## Discussion and conclusions

In tackling Malawi’s high HIV prevalence of 10.6 %, most HIV-prevention interventions in the country have adopted biomedical paradigms, which emphasize the introduction of biologically informed knowledge to reduce risky sexual behaviours [37]. While this approach has been useful in lowering rates of HIV infection, studies have also identified that, even with comprehensive knowledge about HIV transmission, some individuals hold onto myths and misconceptions about HIV transmission [11, 35]. This situation is worrying considering that misconceptions about HIV transmission are associated with various risky behavioural practices [3, 6]. Therefore, the main contribution of this study is its potential to cast light on the factors that give rise to these misconceptions in the first place. Unless these underlying factors are properly identified and addressed, such misconceptions would persist and continue to fuel HIV transmission in spite of improved biological knowledge about the disease.

The finding that a significant proportion of both men and women still hold misconceptions about HIV transmission, including those from the Northern region where literacy levels and HIV/AIDS awareness are remarkably high, suggests that biomedical explanations of HIV transmission with exclusive focus on ABC prevention knowledge may not be sufficient for dispelling misconceptions about HIV transmission [35, 38, 39]. The need for targeted education at eradicating myths and misconceptions about HIV transmission may be emphasized, given that some Malawians report awareness of HIV prevention knowledge, yet still hold on to misconceptions about HIV transmission. However, it is also important that both females and males with higher scores on ABC prevention knowledge were less likely to endorse misconceptions about HIV transmission. These results are consistent with some other studies that suggest that a clear biological and conceptual understanding of HIV transmission can be useful in rejecting misconceptions surrounding the virus [11, 18, 19].

Also, HIV testing has been shown to relate to people’s perception about HIV transmission, based on the counselling and information sharing that occur at the point of testing [17, 40]. This is consistent with our theoretical conceptualization that HIV testing can prove to be an effective strategy for behavioural change by dispelling myths and misconceptions about the virus during counselling [20, 21]. These findings show useful evidence for policymakers to target reduction of misconceptions about HIV transmission with emphasis on HIV/AIDS education to ensure all segments of populations acquire adequate knowledge about HIV transmission and subsequently testing.

The finding that Tumbuka women are more likely to hold misconceptions about HIV transmission than women from other ethnic groups may provide insights into the ethnic interpretations of HIV transmission in Malawi. Compared to other ethnic groups, the Tumbuka, while relatively more literate, adhere strictly to cultural practices and are less likely to participate in HIV reduction practices [23]. This finding is consistent with Munthali [41], who found that the Tumbuka people traditionally believe misfortunes, including illnesses, are caused by their ancestors. Due to this cultural belief, the Tumbuka people do not entirely rely on western medicine and sometimes seek healing by talking to the ancestors to ask what has caused them illness [41]. Moreover, the patriarchal nature of the Tumbuka ethnic group may explain gender variation among the Tumbuka in holding onto misconceptions about HIV transmission [42]. Males belonging to the Tumbuka ethnic group, compared to their female counterparts, may have more access to resources and institutions, which could help in enhancing clinical knowledge and dispelling HIV misconceptions.

It is also noteworthy that after controlling for educational background, the gap in endorsing misconceptions about HIV transmission between Tumbuka women and women from other ethnic groups was widened. This suggests that Tumbuka women are particularly disadvantaged in endorsing such misconceptions when they do not have higher levels of education. Historically, the Tumbuka, compared to other ethnic groups, has had access to formal education through the effort of missionaries associated with the Livingstonia Mission, a branch of the Free Church of Scotland [43]. We argue that there are complex cultural processes and dynamics that fuel endorsing misconceptions about HIV transmission among women in the Tumbuka ethnic group. In-depth qualitative studies may be useful in unpacking the gendered nuances around such misconceptions.

Moreover, we found that Muslim women were more likely to endorse misconceptions about HIV transmission than their Christian counterparts. Empirical findings show that HIV issues are not much discussed because of the strict adherence to religious moral codes that emphasize sexual abstinence [33]. Furthermore, Muslim and other patriarchal societies are characterized by unequal distributions of resources between females and males within households [44]. Thus, compared to Muslim men, Muslim women may experience limited access to resources, including opportunities to obtain clinical knowledge about HIV transmission. It is particularly important for intervention programs to provide such opportunities for Muslim women.

We also observed that male and female respondents in rural areas were more likely to hold misconceptions about HIV transmission than their urban counterparts. These findings are corroborated by other studies, suggesting that rural areas lack access to information and resources to obtain clinical knowledge about HIV transmission [11]. Also, given the rural nature of Northern Malawi [45], it is not surprising that both male and female respondents in the Northern region were more likely to hold misconceptions about HIV transmission than residents of other regions. Higher levels of misconception endorsement could be the factor contributing to an increasing prevalence of HIV in the Northern region [34]. Thus, our findings emphasize the need for intervention programs, particularly in rural areas and the Northern region to provide clinical knowledge about HIV transmission.

As expected, educated and wealthier people were less likely to hold misconceptions about HIV transmission compared with less educated and poorer counterparts in Malawi. These findings are largely consistent with earlier research that indicates that formal education and wealth provide more exposure to knowledge and awareness about HIV transmission for individuals to reject such misconceptions [11, 46]. Thus, although school-based interventions seem to be effective in Malawi, it is still crucial to provide intervention programs to disseminate clinically informed knowledge about HIV transmission, particularly for those who have lower levels of education and limited socioeconomic resources.

This study has some noteworthy limitations. First, the Malawi Demographic and Health Survey (MDHS) is collected contemporaneously, and it is difficult to define causal relationships between dependent and independent variables. Our findings are thus limited to associations. Moreover, qualitative studies show there are other types of misconceptions about HIV transmission in Malawi, such as the belief that sleeping with virgins or making ancestral sacrifices can cure HIV infection [14, 15]. However, our construct of dependent variable is limited to four variables available in the MDHS. Finally, due to the self-reported nature of the MDHS, our results may be biased because of an underreporting issue, particularly as issues regarding HIV are sensitive for respondents. Despite these limitations, our findings make an important contribution to the literature and provide useful information for policymakers in establishing more effective HIV prevention intervention programs in Malawi.

## Abbreviations

ABC, Abstinence, Being faithful, and Condom use, AIDS, Acquired Immune Deficiency Syndrome, HIV, Human Immunodeficiency Virus, MDHS, Malawi Demographic and Health Survey, NGO, Non-Governmental Organisation, OR, Odds Ratio, PCA, Principal Component Analysis, SSA, Sub-Saharan Africa, STIs, Sexually Transmitted Infections
